# Genomic Signature and Mutation Trend Analysis of Pandemic (H1N1) 2009 Influenza A Virus

**DOI:** 10.1371/journal.pone.0009549

**Published:** 2010-03-08

**Authors:** Chungen Pan, Byron Cheung, Suiyi Tan, Chunling Li, Lin Li, Shuwen Liu, Shibo Jiang

**Affiliations:** 1 Lindsley F. Kimball Research Institute, New York Blood Center, New York, New York, United States of America; 2 College of Life Sciences, Peking University, Beijing, China; 3 Southern Medical University, Guangzhou, Guangdong, China; 4 Institute of Veterinary Medicine, Guangdong Academy of Agricultural Sciences, Guangzhou, China; Institut Pasteur, France

## Abstract

A novel swine-origin pandemic influenza A(H1N1) virus (H1N1pdm, also referred to as S-OIV) was identified as the causative agent of the 21^st^ century's first influenza pandemic, but molecular features conferring its ability of human-to-human transmission has not been identified. Here we compared the protein sequences of 2009 H1N1pdm strains with those causing other pandemics and the viruses isolated from humans, swines and avians, and then analyzed the mutation trend of the residues at the signature and non-signature positions, which are species- and non-species-associated, respectively, in the proteins of H1N1pdm during the pandemic of 2009. We confirmed that the host-specific genomic signatures of 2009 H1N1pdm, which are mainly swine-like, were highly identical to those of the 1918 H1N1pdm. During the short period of time when the pandemic alert level was raised from phase 4 to phase 6, one signature residue at the position of NP-100 mutated from valine to isoleucine. Four non-signature residues, at positions NA-91, NA-233, HA-206, and NS1-123, also changed during the epidemic in 2009. All these mutant residues, except that at NA-91, are located in the viral functional domains, suggesting that they may play roles in the human adaption and virulence of 2009 H1N1pdm.

## Introduction

In April 2009, a new influenza A(H1N1) was reported in Mexico and the southwestern United States [1]. The World Health Organization (WHO) boosted its pandemic alert levels for this flu to phase 4 on 27 April 2009, phase 5 on 29 April 2009, and phase 6 on 11 June 2009, declaring a full-blown influenza pandemic for the first time in 41 years. As of 24 January 2010, the laboratory-confirmed cases of pandemic influenza H1N1 2009, including at least 14,711 deaths, had been reported in more than 209 countries and overseas territories or communities worldwide (http://www.who.int/csr/don/2010_01_29/en/index.html).

The causative agent was proven to be a novel swine-origin pandemic influenza A (H1N1) virus (H1N1pdm, also referred to as S-OIV). Its hemagglutinin (HA), nucleoprotein (NP), and nonstructural (NS) protein genes belong to the classical swine lineage, while its neuraminidase (NA) and matrix (M) protein genes derive from a Eurasian swine influenza lineage which entered pigs from avian hosts around 1979, and its polymerase gene segments, PA, PB1 and PB2, descended from the North American triple reassortant swine lineage [2]–[5]. This unique genetic combination may contribute to the improved fitness of the H1N1pdm in humans and its human-to-human transmissibility, although none of the molecular features previously shown to confer increased human-to-human transmissibility has so far been identified in the 2009 H1N1pdm. Since there is a serious concern that the virus may further mutate into a more dangerous form (http://www.cbsnews.com/stories/2009/12/29/health/main6034632.shtml), it is critical to monitor the evolutionary trends of the 2009 H1N1pdm virus.

Shih and colleagues previously developed an entropy-based computational scheme to identify host-specific genomic signatures of human and avian influenza viruses [6]. Most recently, they used this method to compare the protein sequences of the 2009 H1N1pdm strains collected before May 28, 2009, with those of avian, swine and human influenza A viruses (IAVs). Among the 47 avian-human signatures, they found that 8 (one in PB1, one in PB2, 2 in PA and 4 in NP) showed human-characteristic signatures, which may serve as a molecular marker for monitoring adaptive mutations in the influenza viruses [7].

In the present study, we compared the protein sequences of 2009 H1N1pdm strains collected from April 1, 2009 to December 31, 2009, with the corresponding protein sequences of the human, avian, and swine IAVs and those causing past influenza pandemics. We then conducted an analysis to gain insight into 1) the mutation trend of the residues at the signature and non-signature positions in the proteins of H1N1pdm during the pandemic of 2009 and 2) the potential roles of the mutated residues in human adaptation and virulence of the 2009 H1N1pdm influenza virus.

## Results and Discussion

### Comparison of Genomic Signatures of 2009 H1N1pdm with Human, Swine and Avian Influenza A Viruses, as Well as Those of Other Pandemic Influenza Viruses

The consensus protein sequences of the 2009 H1N1pdm were aligned with those of human, avian and swine IAVs collected between 2000 and 2008, as well as those causing past pandemics. The residues in the protein sequences of each group located at the avian-human signature positions described by Chen et al. [7] were listed in Table 1. The signature residues in the proteins of the 2009 H1N1pdm strains collected in the pre-epidemic period were 17%, 94% and 75% identical to those of human, swine and avian IAVs, respectively (Table 2), confirming that the 2009 H1N1pdm originated from swine influenza virus. Notably, the 2009 H1N1pdm exhibited 55%, 15%, 13% and 19% identity of the signatures to the 1918 H1N1pdm, 1957 H2N2pdm, 1968 H3N2pdm and 1977 H1N1pdm, respectively. Similarly, the 1918 H1N1pdm exhibited low similarity of the signatures to human IAVs, while those causing the 1957 H2N2, 1968 H3N2 and 1977 H1N1 pandemics displayed high (>94%) identity of the signatures to human IAVs (Table 2). While these pandemic viruses can all be efficiently transmitted in humans, these results suggest that the 2009 H1N1pdm and 1918 H1N1pdm have much lower human-like signatures than other pandemic influenza viruses, including the 1977 H1N1pdm. Like the 1918 H1N1pdm that had a sister relationship with the “classic” swine H1N1 lineage [8], the 2009 H1N1pdm had closer linkage to swine IAVs than avian and human IAVs. Therefore, it is worthwhile to identify the signature or non-signature residues shared by the 2009 H1N1pdm and 1918 H1N1pdm that may play roles in viral transmission and virulence.

**Table 1:** Comparison of the amino acid signatures in the proteins of 2009 H1N1pdm with those in human, swine, and avian IAVs, as well as those causing past influenza pandemics.

Protein	Position	Amino acid signatures								
		2009 H1N1	1918 H1N1	1957H2N2	1968 H3N2		1977 H1N1	Human	Swine	Avian
PB2	44	A	A	S		S	S	S	A	A
	199	A	A	S		S	S	S	A	A
	***271***	***A***	***T***	***A***		***A***	***A***	***A***	***A***	***T***
	475	L	L	M		M	M	M	L	L
	567	D	N	N		N	N	I	D	D
	588	T	A	I		I	I	I	T	A
	613	V	V	T		T	T	T	V	V
	627	E	K	K		K	K	K	E	E
	702	K	R	R		R	R	R	K	E
PB1	327	R	R	R		K	R	K	R	R
	***336***	***I***	***V***	***V***		***V***	***I***	***I***	***I***	***V***
PA	28	P	L	L		L	L	L	P	P
	55	D	N	N		N	N	N	D	D
	57	R	R	Q		Q	Q	Q	R	R
	100	V	A	A		A	A	A	V	V
	225	S	S	C		C	C	C	S	S
	268	L	L	I		I	I	I	L	L
	***356***	***R***	***K***	***R***		***R***	***R***	***R***	***K***	***K***
	404	A	A	S		S	S	S	A	A
	***409***	***N***	***S***	***N***		***N***	***N***	***N***	***N***	***S***
	552	T	S	S		S	S	S	T	T
NP	16	G	D	D		D	D	D	G	G
	***33***	***I***	***I***	***I***		***I***	***I***	***I***	***I***	***V***
	61	I	I	L		L	L	L	I	I
	***100***	***V***	***I***	***V***		***V***	***V***	***V***	***V***	***R***
	109	I	I	V		V	V	V	I	I
	214	R	R	K		K	K	K	R	R
	283	L	P	P		P	P	P	L	L
	293	R	R	K		K	K	K	R	R
	***305***	***K***	***R***	***K***		***K***	***K***	***K***	***K***	***R***
	313	V	Y	Y		Y	Y	Y	F	F
	***357***	***K***	***K***	***K***		***K***	***K***	***K***	***K***	***Q***
	372	E	E	D		D	D	D	E	E
	422	R	R	K		K	K	K	R	R
	442	T	T	A		A	A	A	T	T
	455	D	D	E		E	E	E	D	D
M1	115	V	V	I		I	I	I	V	V
	121	T	A	A		A	A	A	T	T
	137	T	A	A		A	A	A	T	T
M2	11	T	T	I		I	I	I	T	T
	20	S	N	N		N	N	N	S	S
	57	Y	Y	H		H	H	H	Y	Y
	86	V	V	A		A	A	A	V	V
	93	N	N	S		S	N	S	N	N
NS1	81	I	I	M		M	M	M	I	I
	227	delete	K	R		R	R	R	G	E
NS2	107	L	L	F		F	F	F	L	L

Note: Only the dominant residues were listed. The human-like amino acid signatures in the 2009 H1N1pdm were highlighted in bold and italic.

**Table 2:** The identity of amino acid signatures in the proteins of pandemic IAVs and human, swine, and avian IAVs.

Influenza A virus	Identity (%) of amino acid signatures							
	2009 H1N1	1918 H1N1	1957 H2N2	1968 H3N2	1977 H1N1	Human	Swine	Avian
2009 H1N1pdm	47/47 (100)	26/47 (55)	7/47 (15)	6/47 (13)	9/47 (19)	8/47 (17)	44/47 (94)	35/47 (75)
1918 H1N1pdm	-	47/47 (100)	17/47 (36)	16/47 (34)	18/47 (38)	14/47 (30)	26/47 (55)	30/47 (47)
1957 H2N2pdm	-	-	47/47 (100)	46/47 (98)	45/47 (96)	44/47 (94)	7/47 (15)	2/47 (4)
1968 H3N2pdm	-	-	-	47/47 (100)	45/47 (96)	44/47 (94)	6/47 (13)	1/47 (2)
1977 H1N1pdm	-	-	-	-	47/47 (100)	45/47 (96)	9/47 (19)	1/47 (2)

Unlike seasonal flu that usually hits elderly people the hardest, the 2009 H1N1pdm has mostly infected the young, especially school-aged children [9]. Persons born before 1957 had a reduced risk of 2009 H1N1pdm infection [10], suggesting that the immunity induced by the viruses causing influenza pandemics after 1957 are ineffective in protecting people from infection by the 2009 H1N1pdm. After assessing human sera from different age groups, Itoh et al. [3] found that elderly people exposed to the 1918 H1N1pdm had antibodies that cross-neutralized the 2009 H1N1pdm. Hancock et al. [11] also reported that persons under the age of 30 years had little evidence of cross-reactive antibodies to the 2009 H1N1pdm virus, while people born before 1930, who were probably exposed to a 1918 H1N1pdm-like virus, had the highest titers of antibodies against the 2009 H1N1pdm. These findings suggest that the 2009 and 1918 H1N1pdm viruses have high antigenic and immunogenic similarities, raising serious concerns that the 2009 H1N1pdm may follow an evolutionary path similar to that of the 1918 H1N1pdm.

### Mutation Trend Analysis of the Signature and Non-Signature Residues in Proteins of 2009 H1N1pdm Isolates Collected at the Different Periods in the 2009 Pandemic

**Figure 1:**
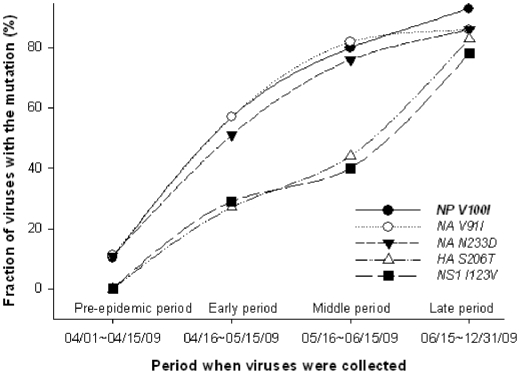
The dominant mutations of the amino acid residues in NP, NA, HA, and NS1 in 2009 H1N1pdm isolates collected from April 1, 2009 to October 25, 2009.

We compared the signature and non-signature residues of the proteins in the 2009 H1N1pdm strains collected at the pre-epidemic, early, middle and late periods of the pandemic in 2009. We found that among the 47 avian-human signatures [7], only one signature residue at position 100 of NP exhibited a dominant change during the 2009 epidemic. In the pre-epidemic period, only 10% of 2009 H1N1pdm strains had valine to isoleucine change at position NP-100, whereas about 57%, 80% and 93% of the virus isolates collected in the early, middle and late periods possessed this change, respectively (Table 3 and Fig. 1), suggesting that this V100I mutation may play some role in the increased transmissibility or infectivity of the 2009 H1N1pdm.Strikingly, the 1918 H1N1pdm also had residue isoleucine at the position NP-100, while other pandemic viruses and human IAVs display valine (Table 3).

**Table 3:** Mutation trend analysis of signature and non-signature amino acid residues in the functional domains of the proteins of 2009 H1N1pdm during the influenza pandemic in 2009.

Viral	Amino	2009 H1N1pdm strains collected during					Dominant residues in proteins of IAV of						
protein	Acid	Change	4/1/09–4/15/09	4/16/09–5/15/09	5/16/09–6/15/09	6/16/09–12/31/09	1918	1957	1968	1977	Human	Swine	Avian
	residue position		pre-epidemic period	early period	middle period	late period	H1N1	H2N2	H3N2	H1N1			
NP	100	V→I	3/29 (10)	231/403 (57)	249/313 (80)	236/254 (93)	I	V	V	V	V	V	R
NA(N1)	91	V→I	4/38 (11)	281/494 (57)	314/383 (82)	486/566 (86)	I	-	-	I	I	I	V
NA(N1)	233	N→D	4/38 (11)	300/584 (51)	303/398 (76)	505/589 (86)	N	-	-	D	N	N	N
HA(H1)	206	S→T	0/43 (0)	139/524 (27)	183/416 (44)	500/601 (83)	S	-	-	S	S	S	S
NS1	123	I→V	0/24 (0)	105/358 (29)	132/330 (40)	199/256 (78)	I	I	I	I	I	I	I

Note: the number in brackets indicates the percent (%) of sequences with the mutated amino acid in total number of the sequences collected in the period; ‘-’ means unavailable in non-H1N1 virus.

**Figure 2:**
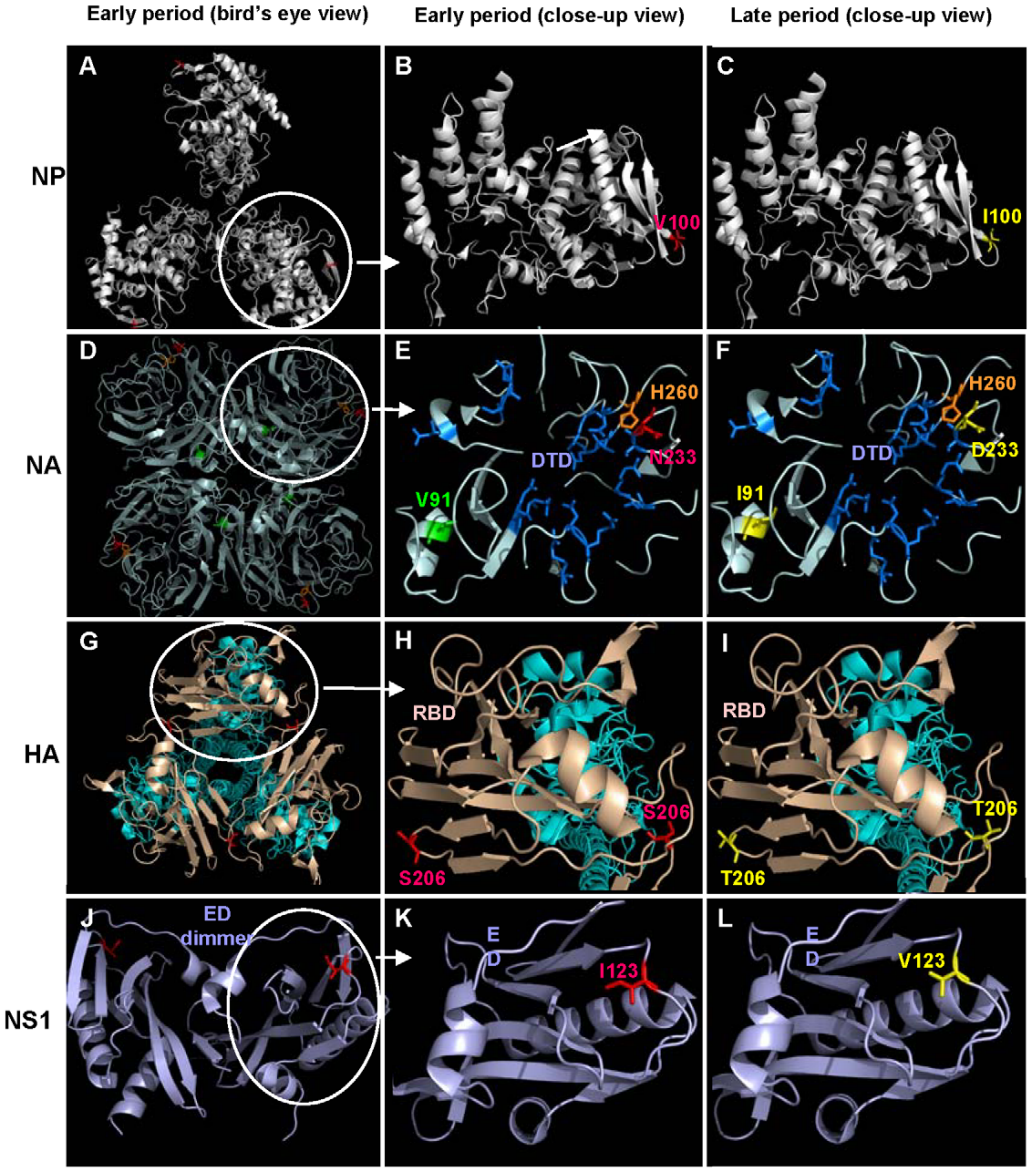
Schematic representation of the functional domains with mutated residues in the 2009 H1N1pdm proteins. **Left panels**: bird's eye view of protein structures of 2009 H1N1pdm collected at the pre-epidemic period in 2009; **Middle panels**: close-up view of the mutated amino acid residues in proteins of 2009 H1N1pdm collected at the pre-epidemic period in 2009; **Right panels**: close-up view of the mutated amino acid residues in proteins of 2009 H1N1pdm collected at the late period in 2009. The amino acid numberings were based on influenza virus A/Puerto Rico/8/1934 (H1N1) [6]. The residues in viruses collected in the pre-epidemic period are colored in red, and those in viruses collected in the late period are colored in yellow. **A–C:** NP trimer and monomer. **D–F:** NA tetramer and monomer. Drug target domain (DTD) is highlighted in dark blue. H260 [274 in A/Vietnam/1203/04(H5N1)] is a critical residue for the NA inhibitor, oseltamivir. NA H274Y mutation results in resistance of 2009 H1N1pdm and other influenza viruses to oseltamivir. **G–I**: HA trimer and monomer. Receptor binding domain (RBD) was highlighted in wheat color, while other part is in green color. **J–L**: Dimer and monomer of effector domain (ED) in NS1.

The influenza viral NP, which forms trimer as a part of the helical genomic ribonucleoprotein complexes, plays a critical role in viral RNA replication [12]. NP may also play a role in cross-species transmission since among the ten IAV proteins, NP contained the largest number (15 of 47) for genomic signatures (Table 1). Each NP monomer, which contains 17 α-helices and 9 β-strands [13], consists of a head domain and a body domain (Fig. 2A). The body domain is comprised of three segments (aa 21–149, 273–396 and 453–489) responsible for binding to the PB1 and PB2 subunits of the viral polymerase [14]. The conserved amino acid regions on the surface of the NP body domain that mediate NP-polymerase interactions are crucial for viral RNA replication. For example, an asparagine to lysine mutation at the position 319, which is located on the surface of the body domain, resulted in an increase of polymerase activity and adaptation of an avian influenza virus to a mammalian host [15]. The residue at NP-100, which is located in the body domain (Fig. 2A–C), is thought to be involved in NP-PB2 interaction [14]. Given that the majority of the viruses gained the V100I mutation during a short period of time when the pandemic alert level was raised from phase 4 to phase 6 (Table 3), this mutation may play a role in the increased transmissibility or infection of the 2009 H1N1pdm.

Furthermore, we identified four dominant mutations of non-signature residues in NA, HA, and NS1 proteins of the 2009 H1N1pdm virus. In NA protein, the avian-like residue, valine at NA-91 mutated to the human-like residue isoleucine, which was presented in the 1918 and 1977 H1N1pdm IAVs. The non-charged residue, asparagine at NA-233, mutated to a negatively charged residue, aspartic acid, which is only presented in the 1977 H1N1pdm IAVs. In the pre-epidemic period, about 11% of the 2009 H1N1pdm strains had the V91I mutation and/or N233D mutation. In the early, middle, and late periods, 57%, 82%, and 86% of the viruses possessed V91I mutation, while 51%, 76% and 86% of the viral isolates had the N233D mutation, respectively (Table 3 and Fig. 1), suggesting that many of the 2009 H1N1pdm strains carried both NA V91I and NA N233D mutations.

In the process of virus infection, NA functions as a tetramer (Fig. 2D) to remove sialic acid from cell-surface receptors to allow the newly made virions to release and spread to uninfected cells [16]. Therefore, NA serves as an important target for development of anti-influenza drugs, such as oseltamivir (Tamiflu) [17] and zanamivir (Relenza) [18]. A single-point mutation of the residues located in the drug target domain (DTD), such as H260Y mutation (corresponding to H274Y mutation in H5N1 viruses), may result in viral resistance to oseltamivir [19]. Most recently, a number of reports indicated that several clinically isolated 2009 H1N1pdm strains with NA H260Y mutation were resistant to the NA inhibitor oseltamivir [20], [21]. However, we did not find the increased H260Y mutation in the 2009 H1N1pdm NA sequences that we analyzed. Instead, we identified V91I and N233D mutations in the majority of 2009 H1N1pdm isolates collected at the late period of the 2009 epidemic. Since the residue at the NA-233 position is also located in the DTD region (Fig. 2E) and has close proximity to H260, it is worthwhile to investigate the potential effect of N233D mutation on the sensitivity of the virus to NA inhibitors. Since the amino acid at NA-91 is not located in the DTD, the NA V91I mutation may have no direct effect on drug sensitivity of the virus.

We identified one dominant mutation in HA, S206T with the mutation rates of 0%, 27%, 44% and 83% in the 2009 H1N1pdm strains collected in the pre-epidemic, early, middle and late periods, respectively (Table 3 and Fig. 1). This is a unique mutation because it was neither found in 1918 and 1977 H1N1pdm viruses, nor was it found in the human, swine and avian IAVs. Interestingly, however, we found that the S206T mutation transiently appeared in the HA sequences of human H1N1 viruses collected in 1934 and in swine H1N1 viruses collected in 1976 and 1977. S206 is located in the receptor-binding domain (RBD) of HA (Fig. 2G-I)[22]. The binding of IAV to erythrocytes and host cells is mediated by the interaction of its HA RBD with the cell surface receptor containing sialic acid. The RBD sequence is thus the major determinant of IAV host specificity [23]; therefore, HA-206 S→T mutation may directly affect the infectivity and transmissibility of 2009 H1N1pdm in humans.

Another unique dominant mutation occurred in the NS1 protein, NS1-123 I→V, during the pandemic in 2009. None of the IAVs collected in the pre-epidemic period carried this mutation, while 29%, 40% and 78% of the 2009 H1N1pdm strains collected in the early, middle and late periods possessed the NS1-123 I→V mutation, respectively (Table 3 and Fig. 1). This dominant mutation has not been observed in other IAVs that caused past influenza pandemics. NS1, a 26-kDa protein, functions as a dimer (Fig. 2J). Its monomer consists of seven β-strands and three α-helices, which form the two functional domains, the RNA-binding groove (RBG) and the effector domain (ED) (Fig. 2K) [24]. NS1 is responsible for suppressing antiviral interferon (IFN) induction during viral replication by preventing activation of the latent transcription factors IRF-3 [25] and NF-κB [26]. The lethal H5N1 strains with a point mutation, D92E, or a deletion of residues 80-84 in the NS1 protein, exhibited increased virulence, cytokine resistance or both [27]. The highly effective 1918 H1N1pdm NS1 protein as an inhibitor of type I IFN production might have contributed to its exceptional virulence [25]. In the 2009 H1N1pdm virus, we did not find D92E or other mutations that confer the high virulence of H5N1 and 1918 H1N1pdm strains. Similarly, none of the previously identified virulence factors, such as PB2-627 E→K mutation [28], [29], has been identified in the 2009 H1N1pdm. Consequently, the potential role of the NS1-123 I→V mutation, which is located in the ED of NS1, in virulence and host adaptation needs to be clarified.

In summary, our study confirms that the 2009 H1N1pdm virus has much closer linkage to the 1918 H1N1pdm than any other pandemic influenza viruses. We identified one dominant mutation at the signature position (NP-100) and four dominant mutations at the non-signature positions (NA-91, NA-233, HA-206, and NS1-123). Except NA-91, all these mutant residues are located in the viral functional domains, suggesting that they may play roles in the human adaption and virulence of 2009 H1N1pdm.

## Methods

### Collection and Analysis of Influenza A Virus Sequences

To compare the protein sequences of the 2009 H1N1pdm with those of other IAVs, we downloaded from the NCBI Influenza Database the full-length or partial protein sequences of the IAVs isolated between 2000 and 2008 from humans (H1N1 and H3N2), avians (H1N1, H3N2, H5N1, and H9N2), and swines (H1N1, H1N2, H2N3, H3N1, H3N2, H3N8, and H4N6, H5N1, and H9N2),), and those causing past and current influenza pandemics, including the 1918 H1N1pdm, 1957 H2N2pdm, 1968 H3N2pdm, 1977 H1N1pdm, and 2009 H1N1pdm. The 2009 H1N1pdm strains were collected from April 1, 2009 to December 31, 2009 (according to NCBI records). The sequences of ten proteins, including PB2, PB1, PA, HA(H1), NA(N1), NP, M1, M2, NS1, and NS2, were analyzed. To monitor the mutation trend of the 2009 H1N1pdm, its protein sequences were divided into 4 groups based on the time the sequences were collected: i) 4/1/09-4/15/09 (pre-epidemic period); ii) 4/16/09-5/15/09 (early epidemic period) when WHO raised the pandemic alert level from 4 to 5; iii) 5/16/09-6/15/09 (middle epidemic period) when WHO raised the pandemic to level 6; and iv) 6/15/09-10/25/09 (late epidemic period). Multiple sequence alignments were performed using an online program (see http://www.ncbi.nlm.nih.gov/genomes/FLU/FLU.html) to obtain the consensus sequences and to identify the dominant mutations in each protein as previously described [30]. A dominant mutation is defined here as one mutated residue containing the largest sequence count compared with other residues at a particular aligned position. All amino acid numberings are based on influenza virus A/Puerto Rico/8/1934 (H1N1) [6].

### Protein Modeling Analysis

Homology-based structural models of the functional domains with or without mutations were constructed with templates downloaded from the Protein Data Bank, including NP (PDB ID: 2IQH), NA (PDB ID: 2HTY), HA (PDB ID: 1RUZ), and NS1 (PDB ID: 3F5T). Briefly, the model of the corresponding protein (e.g., NP) was downloaded from Protein Data Bank and opened with the PYMOL program. The residues in the protein model were replaced with those at the corresponding positions in the protein to be analyzed using the “Mutagenesis” function of PYMOL program [31] (http://www.pymol.org). The main functional domains in the protein were displayed and analyzed.
